# Unilateral, trifocal, diaphyseal fracture of the radius with ipsilateral mid-shaft ulna fracture in an adult: a case report

**DOI:** 10.1186/1752-1947-5-123

**Published:** 2011-03-29

**Authors:** Mazin Ibrahim, Jenny Cwilewicz, Osman H Khan, Anthony Gibbon

**Affiliations:** 1 24 Pinsent Court, York, UK; 2 9 Hambleton Avenue, York, UK; 3 Orthopaedics Department, Pinderfield Hospital, Wakefield, UK; 4 Orthopaedics Department, York Hospital, York, UK

## Abstract

**Introduction:**

To the best of our knowledge, a trifocal, diaphyseal fracture of the radius associated with ipsilateral mid-shaft fracture of the ulna in an adult has not been reported in the literature to date. The AO classification system does not include such a fracture configuration.

**Case presentation:**

We report a case of trifocal, diaphyseal fracture of the radius with a mid-diaphyseal fracture of the ulna in a 53-year-old Caucasian, British, right-hand dominant woman involved in a head-on collision with another vehicle. The management of this rare fracture configuration is described and alternative treatment options discussed.

**Conclusions:**

We describe an unusual, complex fracture, which with prompt surgical treatment resulted in a rapid, full and satisfactory functional recovery for our patient.

## Introduction

Both bone forearm, diaphyseal fractures are commonly encountered in clinical practice. Segmental radius shaft fractures are, however, less commonly seen. We report a case of trifocal, complex diaphyseal fracture of the radius with ipsilateral mid-shaft fracture of the ulna. Our review of the scientific literature revealed no evidence of any previous reports relating to the surgical treatment of such a fracture. However, the management of a trifocal ulna fracture with bifocal radius fracture in a child has been described previously.

## Case presentation

A 53-year-old Caucasian British, right-hand dominant woman was involved in a road traffic accident while driving a car, involving a head-on collision with another vehicle at approximately 30 miles/hour. She sustained a closed injury to the left forearm against the steering wheel, resulting in obvious clinical deformity. No neurovascular deficit was evident.

**Figure 1:**
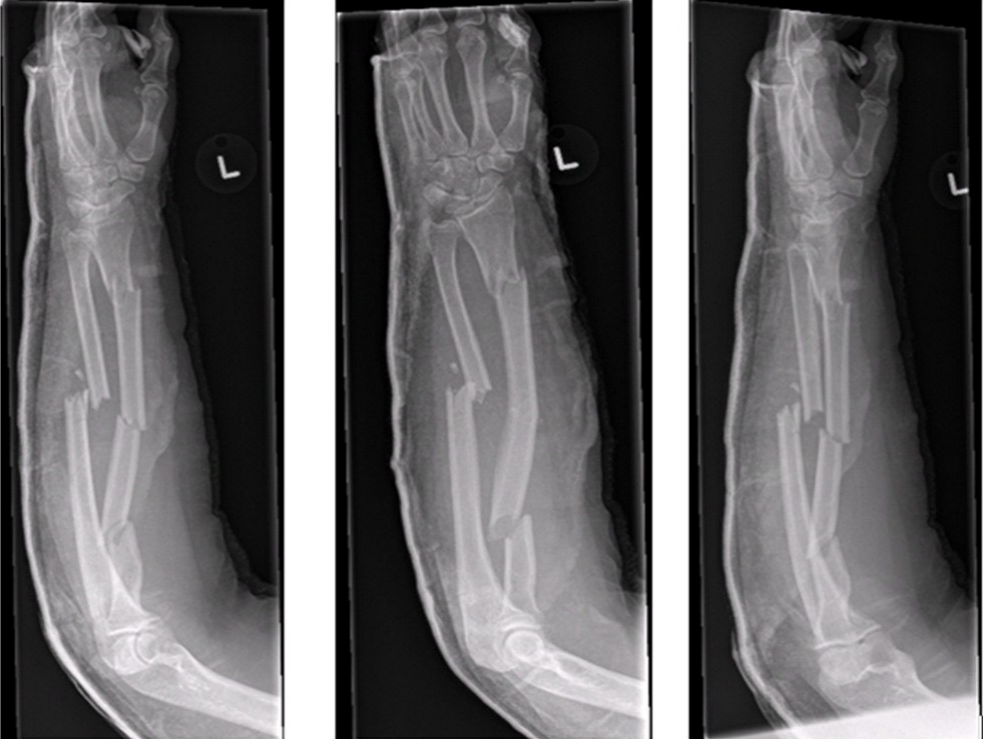
**Left forearm in an above-elbow back slab (anteroposterior and lateral views from the initial injury)**.

Radiographs revealed a displaced and angulated trifocal fracture of the radial shaft in combination with a displaced two-part mid-shaft ulna fracture (Figure 1). Within 24 hours an open reduction and internal fixation of the fracture was performed.

**Figure 2:**
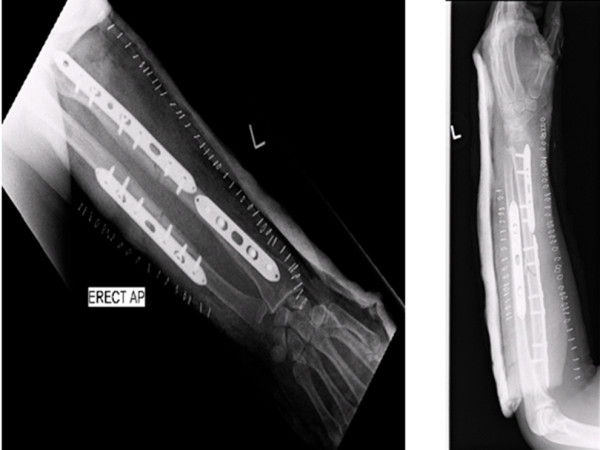
**Open reduction and internal fixation of left radius and ulna with dynamic compression plating (post-operative films)**.

Under general anesthesia, using a direct subcutaneous approach to the ulna, the ulna was reduced and fixed with a seven-hole titanium dynamic compression plate (DCP; Figure 2); 1 mm compression was applied.

The radius was exposed using Henry's approach. The distal radius fracture was fixed using a five-hole titanium DCP while applying 1 mm compression. The proximal three radius fragments were fixed with a nine-hole DCP (Figure 2). Careful handling of the soft tissues was paramount and extra care was taken to avoid devascularising any of the bone fracture segments. We also applied 1 mm compression to the proximal fracture. The middle fracture was bridged because of inherent comminution.

After wound closure, an above-elbow back slab was applied with the elbow held in 90 degrees of flexion. The forearm was held elevated in a sling and our patient was monitored for signs of compartment syndrome. Our patient was discharged from hospital after 48 hours of observation in a broad arm sling; there were no immediate post-operative complications.

After two weeks, the back slab and the skin staples were removed. There was no neurovascular deficit; only a minor but improving subjective altered sensation over the dorsal first web space. The range of active supination was slightly reduced, but otherwise a good range of movement was demonstrated. Our patient was left free of a cast and advised to mobilize her forearm.

At six weeks follow-up, our patient showed further functional improvement. A weakened power grip was noted and physiotherapy initiated. Results as seen on radiographs were satisfactory.

After three months, our patient returned to work as a cashier. She was pain free but reported a weakness in the left forearm and occasional paresthesia over the dorsal first web space.

Our patient completed the Disabilities of the Arm, Shoulder and Hand (DASH) questionnaire and scored 49.1 (measures scaled on a zero to 100 scale: a higher score indicates greater disability). She was finding lifting tasks difficult and did not yet feel able to drive. She had good and equal active and passive range of movement of both wrist and elbow. Grip and pincer strength were measured and values revealed an objective weakness on the left, although this was confounded by dominant limb strength variation.

Our patient's final review took place six months after the initial injury. She had made a complete functional recovery with a full range of movement of elbow and wrist joints, equal on both sides. The altered sensation over the first dorsal web space of the left hand had continued to improve over time. She had resumed driving, remained pain free and her grip strength had been restored.

**Figure 3:**
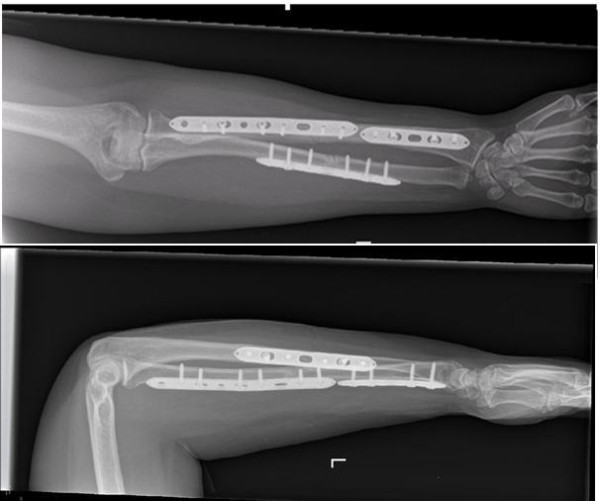
**Left forearm open reduction and internal fixation of radius and ulna at 6-month follow-up (anteroposterior and lateral views)**.

Radiographs revealed that the fractures had united (Figure 3) and our patient was subsequently discharged. No plan was made to remove the plates in the future.

## Discussion

While diaphyseal fractures of the radius and ulna are common, the trifocal diaphyseal fracture of the radius with concurrent mid-shaft ulna fracture is much less frequently encountered. The AO classification system of diaphyseal radius and ulna fractures has described complex fractures of both bones, but only bifocal injuries (that is, involving two points of fracture along a single bone) [1]. No classification system as yet has described this particular type of injury.

Our literature search did not reveal any similar cases. However, there has been a reported case of an ipsilateral diaphyseal fracture of the radius, ulna and radial head [3]. This injury was similarly fixed with DCP plates but our patient also required a radial head replacement. Our patient regained an almost full range of movement.

A closed ipsilateral supracondylar humerus with trifocal ulna and bifocal radius fractures has also been reported [2]. Intra-medullary pinning of diaphyseal fractures of both forearm bones in adults has been found to provide good outcomes [4], as has the use of an interlocking intra-medullary nail, with a mean union time of 15 weeks using an open reduction technique [5].

A retrospective study into 38 cases involving complex fractures of the proximal radius and ulna in adult patients, treated via various methods has been performed. In this study there were seven early revisions due to disassembly of the fixation system, deep infection and insufficient fixation. A number of late complications arose including non-union and malalignment [6]. In cases of non-union of fractures of the radius and ulna there is evidence to suggest that plate fixation with autologous cancellous bone grafting can result in high rates of union and improved upper limb function [7].

In our case, we felt that we could achieve a satisfactory outcome with open reduction and internal fixation using a DCP. The DCP would afford us anatomical reduction, and rigid fixation with rotational control to support fracture union of all segments. There was consideration made of using an intra-medullary nail, but this would not provide either rigid fixation or rotational control, and hence led to non-union, malunion and hence a poor functional outcome. Pre-bent intra-medullary nails with interlocking provide rotational stability and reduce the risks of non-union and malunion. However, their use would be of limited value in controlling a trifocal fracture.

Bone grafting was deemed unnecessary in our case as we managed to reduce the fractures anatomically, under direct compression. The radial bow and length was achieved intra-operatively. We would advocate the use of bone graft in such circumstances where there is marked comminution at fracture ends and where anatomic reduction cannot be achieved. In our patient's case, we also believe that careful preservation of vascularity of the bone fracture segments obviated the need for bone grafting.

Additionally, consideration was made to using a single long DCP plate to fix the segmental radius fracture. However, it would have been extremely difficult to contour the plate. External fixation was deemed inappropriate in the management of this fracture pattern because it would not allow us to achieve anatomic reduction, rigid fixation, and rotational control would have been difficult to achieve.

It is well known that the use of DCP plates causes the phenomenon of 'stress shielding'. In our patient's case, the area of bone between the two radial DCP plates was at potentially higher risk of fracture following another episode of trauma, as a result of 'stress shielding'. Hence, one could advocate the removal of the DCP plates after fracture union. We are not planning to remove the plates; however removal of the distal radial DCP plate would be easier to achieve, with a lower risk of nerve injury, and the proximal plate could remain. Removal of the proximal plate would be associated with a higher risk of nerve injury.

We felt that locked compression plates were not necessary, in view of our patient's good bone quality. However, they would play a useful role in older patients who have poorer bone stock.

This report highlights a rare injury and its successful management. Prompt surgical intervention with the appropriate method of open reduction and internal fixation can lead to a good result.

## Conclusions

The present case report highlights a rare combination of injuries. While such injuries occur infrequently, we should try to obtain anatomical reduction and rigid fixation to achieve the best possible functional outcome, improve the chance of fracture union and possibly reduce the incidence of post-operative complications.

## Consent

Written informed consent was obtained from the patient for publication of this case report and any accompanying images. A copy of the written consent is available for review by the Editor-in-Chief of this journal.

## Competing interests

The authors declare that they have no competing interests.

## Authors' contributions

MI and JC followed up our patient, collected radiograph material, wrote the manuscript and obtained patient consent. MI completed the initial literature search. OK contributed to the discussion and took an editorial role. AG performed the operation and took an editorial role. All authors read and approved the final manuscript.
